# *CYP17 *genetic polymorphism, breast cancer, and breast cancer risk factors: Australian Breast Cancer Family Study

**DOI:** 10.1186/bcr1040

**Published:** 2005-05-12

**Authors:** Jiun-Horng Chang, Dorota M Gertig, Xiaoqing Chen, Gillian S Dite, Mark A Jenkins, Roger L Milne, Melissa C Southey, Margaret RE McCredie, Graham G Giles, Georgia Chenevix-Trench, John L Hopper, Amanda B Spurdle

**Affiliations:** 1 Centre for Genetic Epidemiology, The University of Melbourne, Victoria, Australia; 2 Cancer and Cell Biology Division, Queensland Institute of Medical Research, Herston, Queensland, Australia; 3 Department of Pathology, The University of Melbourne, Parkville, Victoria, Australia; 4 Cancer Epidemiology Research Unit, The Cancer Council of New South Wales, Sydney, New South Wales, Australia; 5 Department of Preventive and Social Medicine, University of Otago, Dunedin, New Zealand; 6 Cancer Epidemiology Centre, The Cancer Council Victoria, Carlton, Victoria, Australia

## Abstract

**Introduction:**

Because *CYP17 *can influence the degree of exposure of breast tissues to oestrogen, the interaction between polymorphisms in this gene and hormonal risk factors is of particular interest. We attempted to replicate the findings of studies assessing such interactions with the -34T→C polymorphism.

**Methods:**

Risk factor and *CYP17 *genotyping data were derived from a large Australian population-based case-control-family study of 1,284 breast cancer cases and 679 controls. Crude and adjusted odds ratio (OR) estimates and 95% confidence intervals (CIs) were calculated by unconditional logistic regression analyses.

**Results:**

We found no associations between the *CYP17 *genotype and breast cancer overall. Premenopausal controls with *A*_2_/*A*_2 _genotype had a later age at menarche (*P *< 0.01). The only associations near statistical significance were that postmenopausal women with *A*_1_/*A*_1 _(wild-type) genotype had an increased risk of breast cancer if they had ever used hormone replacement therapy (OR 2.40, 95% CI 1.0 to 5.7; *P *= 0.05) and if they had menopause after age 47 years (OR 2.59, 95% CI 1.0 to 7.0; *P *= 0.06). We found no associations in common with any other studies, and no evidence for interactions.

**Conclusion:**

We observed no evidence of effect modification of reproductive risk factors by *CYP17 *genotype, although the experiment did not have sufficient statistical power to detect small main effects and modest effects in subgroups. Associations found only in subgroup analyses based on relatively small numbers require cautious interpretation without confirmation by other studies. This emphasizes the need for replication in multiple and large population-based studies to provide convincing evidence for gene–environment interactions.

## Introduction

The association between exposure to endogenous and exogenous steroid hormones and breast cancer risk is well established [1]. Consequently, genetic polymorphisms in genes involved with hormone-metabolizing pathways have been widely studied for evidence of their contribution to breast cancer risk [2,3]. One such candidate gene is *CYP17 *on chromosome 10q24.3, which encodes the enzyme cytochrome P450c17α (17α-hydroxylase; 17/20 lyase). P450c17α functions at two different points in the steroid biosynthesis pathway; the 17α hydroxylase activity can convert progesterone to 17α-hydroxyprogesterone, and the 17/20 lyase function may further convert 17α-hydroxyprogesterone to androstenedione (the precursor of both oestrone and testosterone) [4].

One common polymorphism in *CYP17 *has been extensively studied [5-23]. It is a T→C nucleotide substitution 34 base pairs upstream of the translation initiation site in the 5' promoter region. A subset of the literature refers to the wild-type T allele as *A*_1_, and the variant C allele as *A*_2_. The C allele creates an additional Sp1-type (CCAC**C **box) promoter site, and although it was initially suggested to increase expression of the gene [9], a subsequent study did not observe binding to the human transcription factor Sp-1 [16]. There is conflicting evidence indicating that the *CYP17 *-34T→C polymorphism might influence endogenous steroid hormone levels [11,24-31], and the CC genotype has also been reported to be associated with the relative abundance of the 2OHE and 16α OHE forms of oestrogen [32]. A recent study also found the polymorphism associated with higher levels of DHEAS in premenopausal women and higher levels of oestradiol in postmenopausal women [33].

Although a few studies have found evidence for an association between this polymorphism and risk of breast cancer [7,9,19,23], these positive associations were observed for specific subgroups of cases defined by tumour aggressiveness, age at onset, or family history of breast cancer. Two recent meta-analyses [3,34] showed no overall association of breast cancer with the C (*A*_2_) variant, when comparing allele frequencies, or genotypes defined by these alleles under a dominant or recessive model. Results were consistently null in different ethnic groups [34].

As *CYP17 *may influence the degree of exposure of breast epithelial cells to oestrogen, the possibility that the effects of different hormonal risk factors is dependent on different *CYP17 *genotype is of particular interest. Some studies have suggested that *CYP17 *genotype is associated with hormonal risk factors, and/or that the association between breast cancer and hormonal risk factors depends on *CYP17 *genotypes. That is, *CYP17 *genotype may be an effect modifier. The hormonal risk factors examined in this manner have included age at menarche, age at first birth, use of oral contraceptives, age at menopause, and hormonal replacement therapy. So far, studies examining these gene–environment interactions or effect modifications have generally been small and have reported conflicting results [5,8,9,12,13,15,18,19,21,22,27,35-37]. For example, the *CYP17 *variant was significantly associated with earlier age at menarche in only two of eight reports, and an effect of later age at menarche (at least 13 years) limited to women with the wild-type T (*A*_1_) homozygous genotype was evident in only 4 of 11 reports. Moreover, as alluded to in a recent review of studies of the *CYP17 *polymorphisms and hormone levels [38], the published literature is compromised because many reported 'associations' were not statistically significant, and the possibility of publication bias in selective reporting of such data cannot be excluded.

We previously published Australian data on the overall relationship between this *CYP17 *genetic polymorphism and the risk of breast cancer before the age of 40 years [23]. In the present study of a larger sample of women under 60 years, we considered the issue of effect modification. We studied reproductive and hormone-related factors previously documented to be putative effect modifiers [5,9,11,19,24,35]. We used the same categorization as in the Western New York Breast Cancer Study (WNYBCS) [5], the most comprehensive study assessing CYP17 genotype as a potential effect modifier, in an attempt to replicate findings with an independent data set.

## Materials and methods

### Subjects

The Australian Breast Cancer Family Study (ABCFS) is a population-based case-control-family study of breast cancer before the age of 60 years [39-41]. Sampling of cases was stratified by age at onset, and half were diagnosed before age 40 years, so the study is predominantly of premenopausal women. Cases were women living in Melbourne or Sydney diagnosed with a first primary breast cancer, identified through the Victoria and New South Wales cancer registries. Controls were women with no previous breast cancer selected from the electoral roll (adult registration for voting is compulsory in Australia) by a stratified random sampling, frequency-matched for age. Questionnaires used to measure exposure to risk factors and family cancer history have been described previously [23,40,41]. Ethical approvals for the ABCFS and this genotyping study were obtained from The University of Melbourne, the Cancer Councils of Victoria and New South Wales, and the Queensland Institute of Medical Research.

For the purpose of these analyses, subjects were restricted to the women who identified themselves as being white/Caucasian (details in [23]). Molecular analyses (see [41]) have so far identified 41 Caucasian cases carrying a deleterious germline mutation in either *BRCA1 *or *BRCA2*, and these subjects have been excluded from the analyses.

### Molecular analysis

As described in detail previously [23], the *CYP17 *-34T→C polymorphism was measured in DNA extracted from cases and controls with the use of the AIB1 Prism 7700 Sequence Detection System. DNA was extracted from peripheral blood cells by using salt extraction methods for those recruited before 1995 [42] and with the use of spin columns (Mini blood spin columns; Qiagen, Hilden, Germany) for those recruited from 1995 onwards.

### Statistical methods

As one purpose of this study was to try to replicate the effect modification effects previously reported in the literature that are consistent with an increased exposure to endogenous oestrogen associated with genotypes defined by the C (*A*_2_) variant [5,9,11,19,24,35], we categorized risk factors collected in our study in an identical manner to the most comprehensive study assessing effect modification, the WYNBCS [5]. As far as possible, analyses were adjusted for the same factors and the results presented similarly. Consequently, reproductive variables were categorized as follows: age at menarche (less than 13 years, 13 years or more); age at first birth (less than 25 years, 25 years or more); ever use of oral contraceptives (yes, no); family history of breast cancer (yes, no for any first-degree relative reported to have had breast cancer); age at menopause (less than 48 years, 48 years or more); and ever use of hormone replacement therapy (HRT; yes, no).

The Hardy–Weinberg equilibrium assumption was assessed by comparing the genotype frequencies with those expected on the basis of the observed allele frequencies and random mating by using the Pearson χ^2 ^distribution with one degree of freedom. The associations between risk of breast cancer and risk factors and *CYP17 *genotypes were assessed by multiple linear logistic regression, adjusting for the potential confounders reference age, body mass index, family history, education level, country of birth, benign breast disease, and age at menopause in postmenopausal women. Odds ratios (ORs) and 95% confidence intervals (CIs) were calculated with and without adjustment for measured risk factors. Logistic regression was also used to assess, in controls, the associations of hormone-related risk factors with genotype after adjusting for the above potential confounders. The statistical significance of interaction terms was assessed by the likelihood ratio test. All analyses were conducted with Stata version 8.0. All statistical tests were two-sided and the *P *values quoted are nominal; that is, no attempt was made to adjust for multiple comparisons, either in terms of the number of covariates or in terms of the number of modes of inheritance being considered.

A visual comparison between the results of the ABCFS and those of the WNYBCS was conducted by plotting the corresponding log OR estimates from each study against one another, using R version 1.6.2. The size of the points was proportional to the average of the inverse of standard errors of the two studies for that particular risk factor's estimate, so that larger points were those for which there was more precision. A positive correlation between the data points would be evidence for replication of findings.

## Results

Analyses were conducted for the 1,284 cases and 679 controls genotyped for *CYP17*, including 1,572 premenopausal women (mean age 38.3 years) and 391 postmenopausal women (mean age 52.3 years). There was no evidence of deviation from Hardy–Weinberg equilibrium in controls (*P *= 0.95), but in cases there was marginally significant evidence of such deviation, with a deficiency of heterozygotes (565 observed versus 605.3 expected; *P *= 0.02). The C (*A*_2_) allele frequency in cases was 0.38 (SEM 0.001) in cases, and 0.37 (SEM 0.01) in controls (*P *for difference = 0.8).

Table 1 shows that there were no associations between breast cancer risk and *CYP17 *genotypes under codominant inheritance, among either postmenopausal or premenopausal women, with or without adjusting for covariates. For dominant inheritance, the adjusted OR estimates were 0.94 (95% CI 0.76 to 1.18) and 1.31 (95% CI 0.84 to 2.05) among premenopausal and postmenopausal women, respectively. For recessive inheritance, the adjusted OR estimates were 1.15 (95% CI 0.85 to 1.56) and 1.12 (95% CI 0.60 to 2.09) among premenopausal and postmenopausal women, respectively. We also examined risk for women stratified by report of family history, because our previous examination of a subset of the data found evidence for increased risk associated with the CC (*A*_2_/*A*_2_) genotype among women reporting a first-degree or second-degree family history of breast cancer. The OR for the CC (*A*_2_/*A*_2_) genotype was 1.10 (95% CI 0.81 to 1.49) for no family history and 2.46 (95% CI 0.70 to 8.60) for family history under a codominant model of inheritance, and 1.13 (95% CI 0.85, 1.50) for no family history and 2.37 (95% CI 0.72, 7.79) for family history under a recessive model of inheritance.

We excluded the 41 Caucasian cases with a known *BRCA1 *or *BRCA2 *germline mutation; because these mutations are associated with at least a 10-fold increased breast cancer risk [43], for more than 90% of them the cause of their disease was their germline mutation (that is, less than 10% are likely to be phenocopies). It is possible that their *CYP17 *genotype could have a modifying effect on their disease risk; however, the frequencies of the TT (*A*_1_/*A*_1_), TC (*A*_1_/*A*_2_) and CC (*A*_2_/*A*_2_) genotypes were 41% (*n *= 17), 41% (17) and 18% (7), respectively, in carriers, similar to those of 40% (513), 44% (565), and 16% (206) observed in the non-carrier cases (*P *= 0.95). The C (*A*_2_) allele frequency was 0.38 in both carriers and non-carriers.

Table 2 shows the distribution among controls of the *CYP17 *genotypes defined by the polymorphism, under a dominant model, in relation to reproductive and hormonally related risk factors, and family history status based on first-degree relatives, stratified by menopause status. The only significant association was between age at menarche and *CYP17 *genotype in the premenopausal women (*P *= 0.002), such that controls with the TT (*A*_1_/*A*_1_) genotype were more likely to have an age at menarche of less than 13 years. There was no evidence that women with a T (*A*_1_) allele were more likely to have used HRT. The strengths of the estimated associations between genotype and risk factor were little changed by also adjusting for potential confounders.

Table 3 shows that there was nominally significant evidence that ever use of HRT was associated with an increased risk of breast cancer among all women (OR 1.86; 95% CI 1.11 to 3.12; *P *= 0.02). This effect was significant among women homozygous for the T (*A*_1_) allele (OR 2.40; 95% CI 1.01 to 5.70; *P *= 0.05), but not significant for women with at least one C (*A*_2_) allele (OR 1.93; 95% CI 0.93 to 4.02; *P *= 0.08); the two estimates were not significantly different from one another (*P *= 0.7). There was at best marginally significant evidence that later age at menopause was associated with an increased breast cancer risk in women homozygous for the T (*A*_1_) allele (OR 2.59; 95% CI 0.97 to 6.95; *P *= 0.06), but not in women with at least one C (*A*_2_) allele, and the difference in risk estimate by genotype was also not statistically significant (*P *= 0.06).

**Figure 1:**
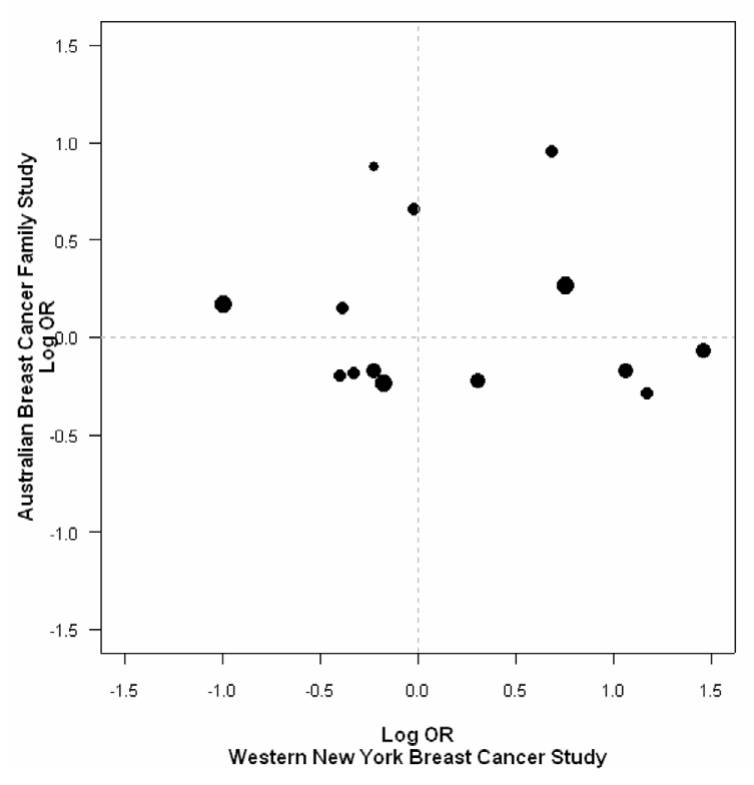
Log odds ratio estimates versus corresponding estimates from the Western New York Breast Cancer Study.

We compared the adjusted OR estimates of each risk factor and breast cancer risk for each *CYP17 *genotype presented in Table 3 with those reported in the literature. A visual comparison between the results of the ABCFS and those of the WNYBCS [5] is shown in Fig. 1. This graphical presentation reveals no association between the point estimates and therefore no evidence for consistency in the estimates overall, or that any one or more findings were strong or statistically significant in both studies. The most consistent finding was for an increased risk in postmenopausal women with older age at menopause and *A*_1_/*A*_1 _genotype, but evidence was weak in the present study and not significant in the WNYBCS [5]. Other studies have examined association between genotype and menopausal status [15] or age at menopause [37], or risk associated with genotype in subgroups stratified by menopausal status [12], and none have reported statistically significant findings. Similarly, we failed to confirm other positive reports of associations with hormonal risk factors, or of effect modification, as detailed below.

## Discussion

Consistent with most previous studies and a recent meta-analysis was our failure to find any evidence for an association between the *CYP17 *genotype defined by the – 34 promoter region T→C nucleotide-substitution polymorphism and risk of breast cancer overall, or within premenopausal or postmenopausal women, whether the genotype be defined under a codominant or a recessive mode of inheritance. Specifically, we failed to confirm our own positive finding [23] of a significantly increased risk associated with the CC (*A*_2_/*A*_2_) genotype in women reporting a positive family history.

When we examined associations between *CYP17 *genotypes and hormone-related risk factors in controls, the only significant finding was an increased risk for older age at menarche among premenopausal women with the C (*A*_2_) allele. This finding is not consistent with previous positive reports, or with the hypothesis that the C (*A*_2_) allele is associated with increased endogenous oestrogen levels and an earlier age at menarche. The point estimates for the effects on breast cancer risk of later age at menopause and HRT use were stronger among women carrying the TT (*A*_1_/*A*_1_) genotype, but the interaction terms were not statistically significant.

Other studies investigating the influence of *CYP17 *genotype on hormonal risk factors have found varying results. For example, the *CYP17 *variant was associated with earlier age at menarche in only two of eight reports [8,9,13,18,21,27,36,37]. Positive association with early age at first birth was observed in two of three reports [5,8,9,11,21], and only single studies have reported significant associations with decreased use of HRT [35] and with decreased difficulty in becoming pregnant [5].

Results from previous studies on the effect modification of reproductive risk factors by *CYP17 *genotypes have also been conflicting. The most consistent association reported was an effect modification of age at menarche. Four studies have presented evidence that the protective effect for a later age at menarche (at least 13 years) was mainly limited to women with the wild-type homozygous genotype [5,9,11,19], but in two of these studies the effect was observed only in premenopausal women [5,19], and seven other studies have failed to confirm these results [8,12,13,18,21,27,36]. Reports of significant associations between risk and age at first birth within strata of *CYP17 *genotype were in opposing directions from two studies reporting such results [11,21].

Our larger study found no association between breast cancer and age at menarche or age at first birth within any of the variant genotypes, either overall or within postmenopausal or premenopausal women. Although we have found at best marginally significant associations between breast cancer risk and both HRT use and age at menopausal status within some *CYP17 *genotype groups, these must be interpreted with caution. We considered both premenopausal and postmenopausal women, seven risk factors, and two genotype groups, so by chance alone we would expect to find a few significant results even if there were no real effects.

Perhaps more convincing evidence against a role of this *CYP17 *variant in modulating endogenous oestrogen levels and associated breast cancer risk factors is the results of a recent study of 1,975 postmenopausal women, which found no association between *CYP17 *genotypes defined by several polymorphisms and mean levels of sex hormones, in particular oestradiol, oestrone and sex-hormone-binding globulin [25]. This suggests that there might be little if any functional effect of the common *CYP17 *polymorphisms, at least among postmenopausal women, although there is a possibility that there might exist other variants and haplotypes associated with hormone levels and risk of breast cancer.

Although we have conducted a relatively large study, there are some limitations. Because the functionality, if any, of the polymorphism we have studied is not well established, it was not possible to specify *a priori *hypotheses about the likely existence and direction of interactions with risk factors. However, the few positive associations with serum hormone levels reported in the literature would suggest that the C (*A*_2_) variant would be associated with increased endogenous hormone levels, and we have observed significant effects both contradicting and in support of such an association. In addition, in this and other similar studies, there are multiple tests being conducted; the quoted *P *values are only nominal and should be interpreted accordingly. Consequently, we cannot claim with confidence that any of our 'significant' findings represent true effects. As there seems to be no overall effect of *CYP17 *genotype on breast cancer risk, finding any true interactions with breast cancer risk factors (should they exist) will require massive individual studies, or pooling of studies. The marginally significant deviation from Hardy–Weinberg equilibrium is unlikely to be due to genotyping error because cases and controls were genotyped at the same time on the same PCR plates, and thus any genotyping bias (and deviation from Hardy–Weinberg equilibrium) would be expected to be seen equally in both cases and controls, but this was not so. Furthermore, our PCR success rate was more than 99.5% for both cases and controls, and results were fully concordant for a subset of 168 duplicate DNAs for which PCR was successful.

The lack of significant associations in our data could be a consequence of not having sufficient statistical power to detect real effects. In terms of detecting a real effect on breast cancer risk associated with the homozygote *A*_2_/*A*_2 _(CC) genotype (whose frequency in controls is 14%; see Table 1), with the total sample sizes we studied we would have had 80% power at the 0.05 level of statistical significance to detect effects greater than the threshold of 1.5-fold. If analyses were restricted to postmenopausal women, this detection threshold would become about twofold, whereas if we were to consider only women with a family history the threshold would be about threefold to fourfold. Within the two genotype groups (for example *A*_1_/*A*_1 _compared with *A*_1_/*A*_2 _and *A*_2_/*A*_2_, which subdivides controls 39:61; see Table 1), the detection thresholds for effects associated with the risk factors (most of which are divided about 40:60 into two groups; see Table 3) would be a minimum of 1.8-fold, and much greater for the smaller subgroupings.

Our study sample was in general younger than that of the other studies reporting on possible effect modification by *CYP17 *genotype. Given that the younger the age at onset of breast cancer the stronger are the familial effects [41], one might expect the effects of genetic factors to be more pronounced in earlier onset disease. We therefore interpret our essentially null results as further support for the increasing body of evidence suggesting that there are no true associations or effect modifications, or at most weak ones, associated with this specific genetic variant of *CYP17*.

## Conclusion

In summary, there are no known data to support a functional effect of this *CYP17 *polymorphism, and although lack of a demonstrated association with serum sex hormones does not exclude a possible functional effect, it does decrease enthusiasm for a possible modifying role of this *CYP17 *polymorphism on breast cancer risk. We have found little evidence to support previous reports of gene–environment interaction, in particular those of the most comprehensive study assessing relationships between *CYP17 *genotype and breast cancer hormonal risk factors [5]. Our post hoc power calculations show that we cannot exclude small main effects, or modest effects within subgroups. It is sobering to note that, if the aim of a study is to detect interactions, the size of the study will have to be at least four times larger than if attention were confined to detecting main effects of the same magnitude [44]. Given the concomitant issues of multiple testing, very large studies of tens of thousands of subjects will be required to evaluate gene-environment interactions, should they exist, and these will probably require the pooling of data from multiple studies (such as has been done by the Collaborative Group on Hormonal Factors in Breast Cancer).

Despite early enthusiasm and numerous reports of positive associations between genotypes, risk factors and breast cancer risk from small studies, few of these have held up under further scrutiny through being reproduced in larger studies. One educational example is of the association between breast cancer and a protein-truncating variant in CHK2 carried by 2.1% of women. Even in a study of more than 10,000 cases and 9,000 controls reporting a 2.3-fold increased risk for the CHK2 mutation and a 1.4-fold increased risk of family history, no nominally significant interactions were found between CHK2 genotypes and family history (*P *= 0.1) [45]. Future studies evaluating gene–environment interaction and cancer risk will need to be very large to produce credible evidence.

## Abbreviations

ABCFS = Australian Breast Cancer Family Study; CI = confidence interval; HRT = hormone replacement therapy; OR = odds ratio; PCR = polymerase chain reaction; WNYBCS = Western New York Breast Cancer Study.

## Competing interests

The author(s) declare that they have no competing interests.

## Authors' contributions

J-HC conducted the analyses and led the manuscript preparation. DMG led the literature review and interpretation of the data. XC conducted the genotyping assays. GSD was responsible for data management. MAJ, RLM and JLH were responsible for development and supervision of the statistical analyses. MCS was responsible for the management of laboratory staff and biospecimens and participated in the quality control of the genotyping and data cleaning. MREMcC, GGG and JLH initiated the ABCFS and have been instrumental in the ongoing execution of these studies. GC-T initiated and obtained funding for the genotyping component of this study, and was involved in the supervision of laboratory work. ABS was responsible for designing and supervising the genotyping and laboratory work, genotype data cleaning, literature review and manuscript preparation. All authors read and approved the final manuscript.

## Figures and Tables

**Table 1:** *CYP17 *genotypes and breast cancer risk by menopausal status (*BRCA1 *and *BRCA2 *mutation carriers excluded)

Genotype	Cases, *n *(%)	Controls, *n *(%)	Crude OR (95% CI)	Adjusted OR (95% CI)^a^
Premenopausal				
*A*_1_/*A*_1 _(TT)	414 (40)	201 (38)	1.00	1.00
*A*_1_/*A*_2 _(TC)	460 (44)	253 (48)	0.88 (0.70, 1.11)	0.90 (0.71, 1.14)
*A*_2_/*A*_2 _(CC)	169 (16)	75 (14)	1.09 (0.79, 1.51)	1.08 (0.78, 1.51)
Postmenopausal				
*A*_1_/*A*_1 _(TT)	99 (41)	66 (44)	1.00	1.00
*A*_1_/*A*_2 _(TC)	105 (44)	64 (43)	1.09 (0.70, 1.70)	1.32 (0.82, 2.12)
*A*_2_/*A*_2 _(CC)	37 (15)	20 (13)	1.23 (0.66, 2.31)	1.29 (0.66, 2.52)
Pooled				
*A*_1_/*A*_1 _(TT)	513 (40)	267 (39)	1.00	1.00
*A*_1_/*A*_2 _(TC)	565 (44)	317 (47)	0.93 (0.76, 1.14)	0.96 (0.77, 1.18)
*A*_2_/*A*_2 _(CC)	206 (16)	95 (14)	1.13 (0.85, 1.50)	1.12 (0.83, 1.51)
No family history^b^				
*A*_1_/*A*_1 _(TT)	462 (40)	249 (39)	1.00	1.00
*A*_1_/*A*_2 _(TC)	501 (44)	296 (47)	0.91 (0.74, 1.13)	0.95 (0.76, 1.18)
*A*_2_/*A*_2 _(CC)	182 (16)	91 (14)	1.08 (0.80, 1.45)	1.10 (0.81, 1.49)
With family history^b^				
*A*_1_/*A*_1 _(TT)	51 (37)	18 (42)	1.00	1.00
*A*_1_/*A*_2 _(TC)	64 (46)	21 (49)	1.08 (0.52, 2.23)	1.08 (0.49, 2.36)
*A*_2_/*A*_2 _(CC)	24 (17)	4 (9)	2.12 (0.65, 6.94)	2.46 (0.70, 8.60)

^a^Odds ratio (OR) adjusted for reference age, body mass index, family history defined by any first-degree relative who had breast cancer, state, education level, country of birth, benign breast lump and age at menopause in postmenopausal women.

^b^Family history defined by any first-degree relative who had breast cancer.

**Table 2:** Breast cancer risk factors by *CYP17 *genotype (*BRCA1 *and *BRCA2 *mutation carriers excluded)

Factor	Premenopausal (*n *= 529)		Postmenopausal (*n *= 150)		Pooled (pre- and postmenopausal) (*n *= 679)	
*CYP17 *genotype...	*A*_1_/*A*_1_	*A*_1_/*A*_2 _and *A*_2_/*A*_2_	*A*_1_/*A*_1_	*A*_1_/*A*_2 _and *A*_2_/*A*_2_	*A*_1_/*A*_1_	*A*_1_/*A*_2 _and *A*_2_/*A*_2_
Age at menarche						
<13 years	95 (48)	112 (34)**	28 (42)	35 (42)	123 (46)	147 (36)***
≥13 years	105 (52)	216 (66)	38 (58)	49 (58)	143 (54)	265 (64)
Age at first birth						
<25 years	52 (37)	93 (40)	37 (61)	40 (54)	89 (44)	133 (43)
≥25 years	89 (63)	142 (60)	24 (39)	34 (46)	113 (56)	176 (57)
Ever use oral contraceptives						
No	12 (6)	20 (6)				
Yes	189 (94)	308 (94)				
Family history of breast cancer						
No	189 (94)	309 (94)	60 (91)	78 (93)	249 (93)	387 (94)
Yes	12 (6)	19 (6)	6 (9)	6 (7)	18 (7)	25 (6)
Age at menopause						
<48 years			40 (61)	45 (54)		
≥48 years			26 (39)	39 (46)		
Ever use HRT						
No			28 (42)	33 (48)		
Yes			38 (58)	51 (61)		

Data are presented as *n *(%). **P = 0.002, ***P = 0.006. Information on difficulty getting pregnant was unavailable. HRT, hormone replacement therapy.

**Table 3:** Breast cancer risk and risk factors by *CYP17 *genotype (*BRCA1 *and *BRCA2 *mutation carriers excluded)

Factor	All data			*A*_1_/*A*_1_			*A*_1_/*A*_2 _and *A*_2_/*A*_2_		
	Case, *n *(%)	Control, *n *(%)	OR (95% CI)	Case, *n *(%)	Control, *n *(%)	OR (95% CI)	Case, *n *(%)	Control, *n *(%)	OR (95% CI)
Premenopausal									
Age at menarche									
<13 years	435 (42)	207 (39)	1.00	183 (45)	95 (47)	1.00	252 (40)	112 (34)	1.00
≥13 years	603 (58)	321 (61)	0.93 (0.7–1.2)	228 (55)	105 (53)	1.18 (0.8–1.8)	375 (60)	216 (66)	0.79 (0.6–1.1)
Age at first birth									
<25 years	318 (40)	145 (39)	1.00	131 (42)	52 (37)	1.00	187 (39)	93 (40)	1.00
≥25 years	479 (60)	231 (61)	1.13 (0.9–1.5)	183 (58)	89 (63)	0.93 (0.6–1.5)	296 (61)	142 (60)	1.30 (0.9–1.8)
Ever use OC									
No	87 (8)	32 (6)	1.00	32 (8)	12 (6)	1.00	55 (9)	20 (6)	1.00
Yes	955 (92)	497 (94)	0.82 (0.5–1.4)	382 (92)	189 (94)	0.75 (0.3–1.9)	573 (91)	308 (94)	0.82 (0.4–1.7)
Postmenopausal									
Age at menarche									
<13 years	101 (42)	63 (42)	1.00	44 (44)	28 (42)	1.00	57 (40)	35 (42)	1.00
≥13 years	140 (58)	87 (58)	0.92 (0.6–1.5)	55 (55)	38 (58)	0.83 (0.4–1.9)	85 (60)	49 (58)	0.84 (0.4–1.7)
Age at first birth									
<25 years	122 (60)	77 (57)	1.00	47 (57)	37 (61)	1.00	75 (62)	40 (54)	1.00
≥25 years	79 (40)	58 (43)	0.98 (0.6–1.6)	35 (43)	24 (39)	1.16 (0.5–2.7)	44 (38)	34 (46)	0.84 (0.4–1.7)
Ever use HRT									
No	79 (33)	61 (41)	1.00	31 (31)	28 (42)	1.00	45 (32)	33 (39)	1.00
Yes	165 (67)	89 (59)	1.86 (1.1–3.1)^1^	68 (69)	38 (58)	2.40 (1.0–5.7)^2^	97 (68)	51 (61)	1.93 (0.9–4.0)^3^
Age at menopause									
<48 years	136 (56)	85 (57)	1.00	50 (51)	40 (61)	1.00	83 (58)	45 (37)	1.00
≥48 years	107 (44)	65 (43)	1.25 (0.7–2.2)	48 (49)	26 (39)	2.59 (1.0–7.0)^4^	59 (42)	39 (63)	0.80 (0.4–1.7)

Adjusted odds ratios (ORs) for premenopausal women are adjusted for age at menarche, age at first birth, ever use of OC, reference age, body mass index, family history defined by any first-degree relative who had breast cancer, state, education level, country of birth and benign breast lump disease. The same adjustment was performed for postmenopausal women except for ever use of hormone replacement therapy (HRT) instead of ever use of OC; adjustment was also made for age at menopause. ^1^*P *= 0.02, ^2^*P *= 0.05, ^3^*P *= 0.08, ^4^*P *= 0.06.
